# Using machine learning to predict subsequent events after EMS non-conveyance decisions

**DOI:** 10.1186/s12911-022-01901-x

**Published:** 2022-06-23

**Authors:** Jani Paulin, Akseli Reunamo, Jouni Kurola, Hans Moen, Sanna Salanterä, Heikki Riihimäki, Tero Vesanen, Mari Koivisto, Timo Iirola

**Affiliations:** 1 Department of Clinical Medicine, University of Turku and Turku University of Applied Sciences, Turku, Finland; 2 Department of Biology, University of Turku, Turku, Finland; 3 Centre for Prehospital Emergency Care, Kuopio University Hospital and University of Eastern Finland, Kuopio, Finland; 4 Department of Computing, University of Turku, Turku, Finland; 5 Department of Nursing Science, University of Turku and Turku University Hospital, Turku, Finland; 6 Department of Nursing Science, University of Turku, Turku, Finland; 7 Department of Biostatistics, University of Turku, Turku, Finland; 8 Emergency Medical Services, Turku University Hospital and University of Turku, Turku, Finland

**Keywords:** Emergency medical service, Non-conveyance, Subsequent event, Patient safety, Machine learning, Text classification, Documentation

## Abstract

**Background:**

Predictors of subsequent events after Emergency Medical Services (EMS) non-conveyance decisions are still unclear, though patient safety is the priority in prehospital emergency care. The aim of this study was to find out whether machine learning can be used in this context and to identify the predictors of subsequent events based on narrative texts of electronic patient care records (ePCR).

**Methods:**

This was a prospective cohort study of EMS patients in Finland. The data was collected from three different regions between June 1 and November 30, 2018. Machine learning, in form of text classification, and manual evaluation were used to predict subsequent events from the clinical notes after a non-conveyance mission.

**Results:**

FastText-model (AUC 0.654) performed best in prediction of subsequent events after EMS non-conveyance missions (n = 11,846). The model and manual analyses showed that many of the subsequent events were planned before, EMS guided the patients to visit primary health care facilities or ED next or following days after non-conveyance. The most frequent signs and symptoms as subsequent event predictors were musculoskeletal-, infection-related and non-specific complaints. 1 in 5 the EMS documentation was inadequate and many of these led to a subsequent event.

**Conclusion:**

Machine learning can be used to predict subsequent events after EMS non-conveyance missions. From the patient safety perspective, it is notable that subsequent event does not necessarily mean that patient safety is compromised. There were a number of subsequent visits to primary health care or EDs, which were planned before by EMS. This demonstrates the appropriate use of limited resources to avoid unnecessary conveyance to the ED. However, further studies are needed without planned subsequent events to find out the harmful subsequent events, where EMS non-conveyance puts patient safety at risk.

## Background

Non-conveyance by Emergency Medical Services (EMS) is a key element in reducing the workload for Emergency Departments (ED) [1]. Globally the non-conveyance rates vary from 3.7 to 93.7% [2] and in Finland, the rate is around 40% [3–5]. The decision to discharge the patient at the scene is complex and is influenced by many factors [2]. Under-triage is a threat to patient safety, but over-triage wastes the limited resources [6]. Many of the subsequent events are related to difficulties in clinical judgment [7]. EMS arrival time at night [8], older age and abnormal vital signs, for example, have been found to predict a subsequent event [8, 9]. However, patient safety is the priority in prehospital emergency care [1, 7]. Machine learning has been seen as a promising method to improve the practice of health care [10], as previous studies have shown that artificial intelligence can be used to identify high risk patients [11–14].

In this study, the focus is to leverage natural language processing (NLP) and machine learning to computationally analyze the narrative texts of electronic patient care records (ePCR) in relation to non-conveyance decisions. Machine learning models are performance focused; they are powerful predictors, but the underlying reasons for their predictions are often not transparent, especially for more complex models. Thus, multiple different model explainability or explainable artificial intelligence (XAI) techniques have been developed to shed more light on what happens inside such complex models [15]. Different text classification models are used in the presented work. A model explainability technique is used as a means to calculate the importance of the input features (words in our case) relative to the predictions made by the model.

The Local Interpretable Model-agnostic Explanations (LIME) method is a popular model explainability technique developed by Ribeiro et al. [16], which uses ridge regression to create a locally fateful simpler model to explain single predictions made by a complex model. Coefficients of ridge regression are used as an importance measure of the input features (words in our case). LIME is model agnostic, thus it can be used to explain any model. This feature was important for us since the best performing model for classification of narrative texts of ePCRs is unknown.

Finally, the safety factors of EMS non-conveyance and the following subsequent events are unclear [2, 17, 18]. To the best of our knowledge, there are no existing studies that report on the use of machine learning to analyze narrative texts of EMS ePCRs. The aim of this study was to find out whether machine learning can be used in this context and to identify the predictors of subsequent events after a non-conveyance decision based on EMS care providers’ documentation. Machine learning in the form of a text classification algorithm was used to predict the events in terms of subsequent event or not, and the LIME model explainability technique and manual evaluation were applied to shed light on possible commonalities between the cases.

## Methods

### Design

This is a prospective cohort study.

### EMS in Finland

The emergency number 112 is in use for all emergencies and there are six regional emergency medical communication centers (EMCCs) administered by a national dispatch authority. After criteria-based protocol, medical calls are prioritized into four categories A to D, where A is the most urgent one.

In Finland, EMS is provided by the hospital districts and is a part of specialized care. Advanced Life Support units (ALS) with at least one paramedic-nurse with 4 years bachelor-level education are the most common ones. EMS units operate typically 24/7. A non-conveyance decision is made based on standing order or by consulting EMS or primary care physician. Depending on the patients’ needs, the patients may be conveyed to central or regional hospitals, municipal healthcare centers or other primary care units by EMS personnel. When needed, patients are conveyed to university hospitals located in neighboring areas. Later in the text, the term ED (Emergency Department) refers to hospitals and specialized medical care and the term primary health care facility refers to primary care units.

### Data

**Fig. 1:**
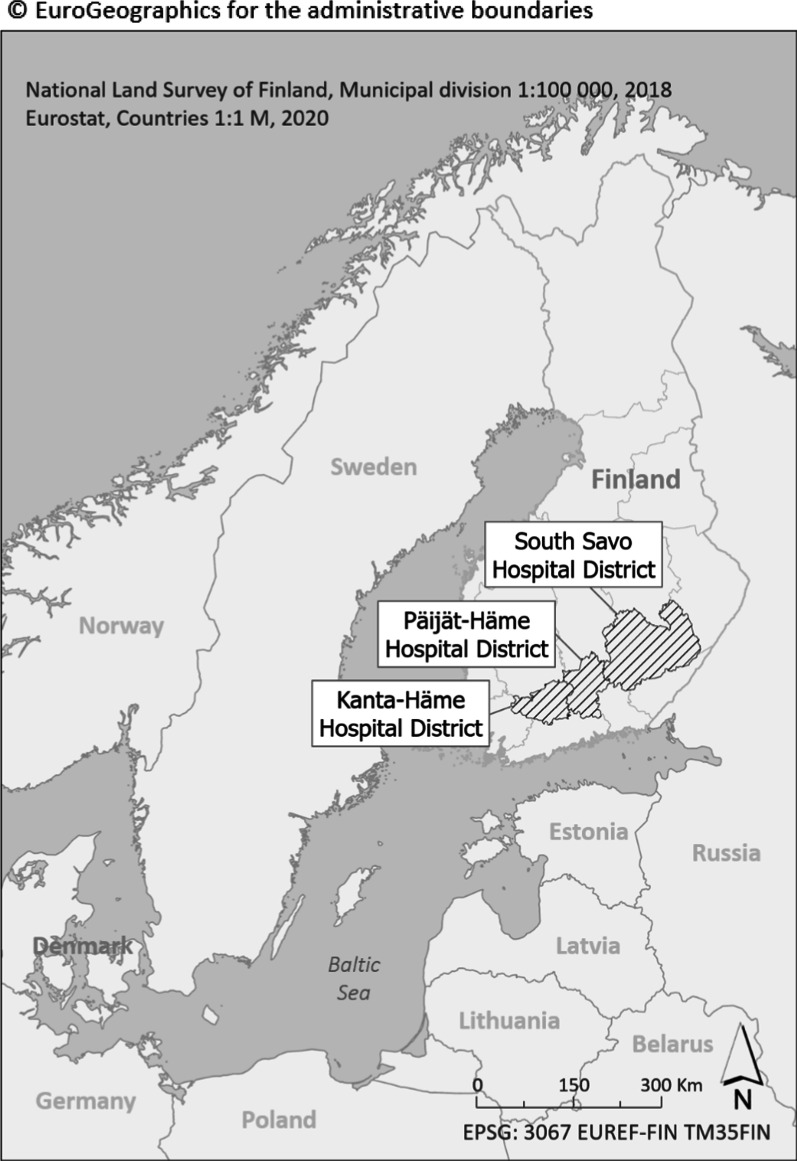
Study areas (published with permission, Paulin et al. [8])

The EMS data from the hospital districts of South-Savo, Kanta-Häme, and Päijät-Häme were collected between June 1 and November 30, 2018 (Fig. 1). The study area comprises both urban and rural areas, with a total of 32 municipalities. Altogether 482,805 inhabitants live in this area, which amounts to 8.8% of the Finnish population. The average population density is 26.1 people per square kilometer.

**Fig. 2:**
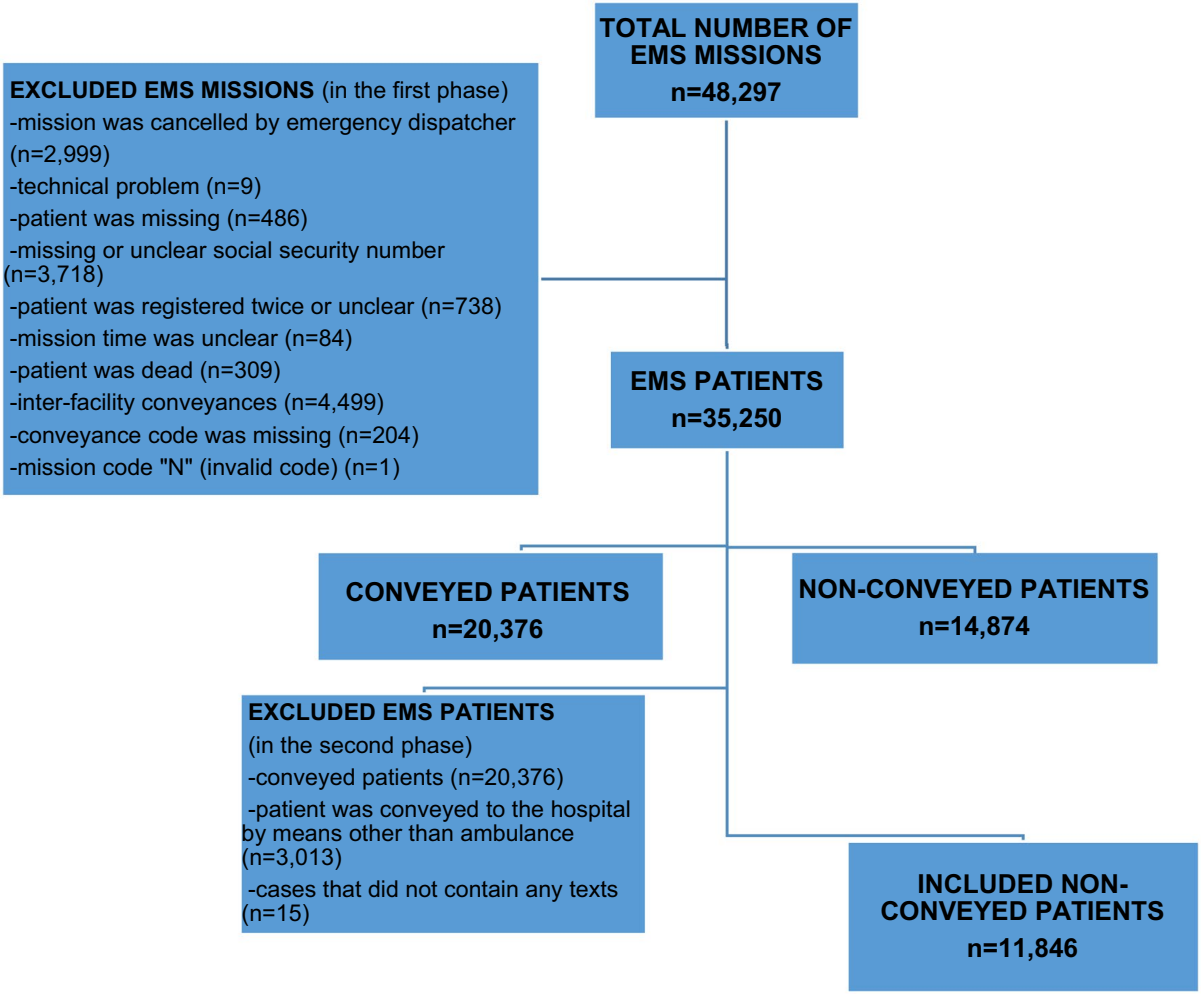
Flow chart

The non-conveyed patients (n = 11,861), which were discharged at scene by EMS after assessment and treatment, were included in the analyses (Fig. 2). The patients were identified using unique 10-digit personal identity numbers and linked between registries. The more detailed description of the data collection, registries, non-conveyed patients and the rates of subsequent events (EMS re-contacts, primary health care facility or ED visits and hospitalization within 48 h and 28 days mortality) after the non-conveyance missions were described previously [3, 8].

**Fig. 3:**
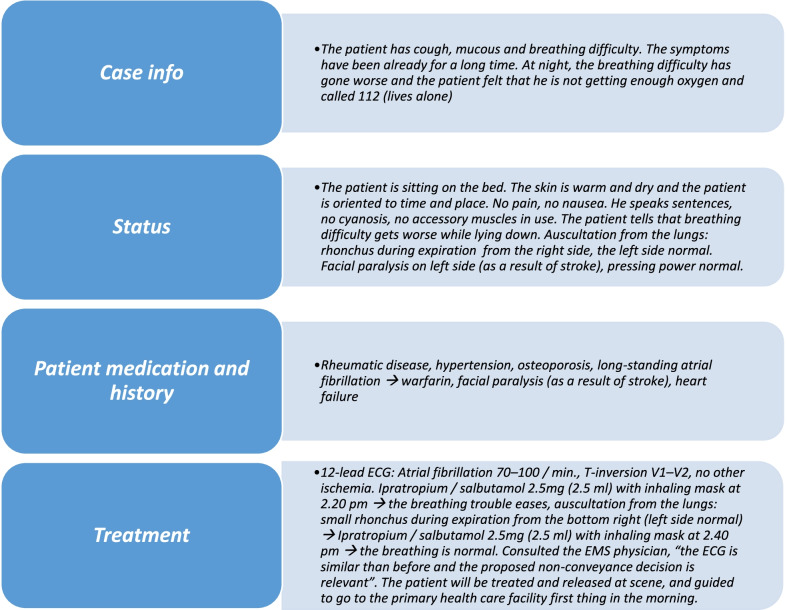
An example of EMS notes

The data consists of the narrative texts of ePCRs of the non-conveyed patients including the scenario, status, previous diseases and medication, treatment, additional information and the reasoning for the non-conveyance decision. Each sample of individual care events contains also a unique identifier and information about the following events. The data from each patient was labeled with the labels: 0—“non-subsequent event”, or 1—“subsequent event”. As part of the preprocessing, all text was lowercase and special characters were removed (all other characters were removed that were not alphabetical (UTF-8) or numbers). In total, the data consisted of 11 861 non-conveyed patients where 9 308 patients belonged to a class non-subsequent event (label 0) and 2553 belonged to a class subsequent event (label 1). Finally, samples that did not contain any text were discarded resulting in a dataset of 11 846 samples where 9 296 patients belonged to the class “non-subsequent event” (label 0) and 2 550 belonged to the class “subsequent event” (label 1). In total, data contains 1.17 million tokens. The minimum number of tokens is four and the maximum 479, and the median number of tokens is 94. Figure 3 provides an example of the narrative text of ePCR without abbreviations and structured data.

### Experimental setup

First, a performance evaluation was done for three candidate text classification models; long short-term memory-model (LSTM-model) with one LSTM-layer [19], Bidirectional-LSTM-model with two LSTM-layers [20] and FastText-model [21]. LSTM-model and Bidirectional-LSTM-model are neural networks that contain LSTM cells; regular LSTM cells are unidirectional and bidirectional-LSTM cells include negative time direction [19, 20]. LSTM models are improved versions of recurrent neural networks and they are well suited for sequential data [22]. FastText uses word and word n-gram embeddings to create text embeddings which are used as input for a linear classifier [21]. Even though FastText is a lot simpler than non-linear LSTM-based models, it has been shown to be almost as good as neural network-based methods in text classification [21]. These models were chosen as candidates to evaluate performance in different model complexity levels. An additional reason to select these models for the experiment was their good performance in a similar task where the same models were used to classify sentences extracted from nursing entries to the right subject [23]. Traditional machine learning setup, splitting data to training, validation, and test set would have not been able to provide a realistic estimate of the performance, because a small test set is not able to describe true data distribution. Thus, fivefold nested cross-validation with five parameters was used to obtain as unbiased and robust evaluation of the performance as possible. Nested cross-validation enables a simple and effective way to select models and evaluate performance [24]. LSTM-model and Bidirectional-LSTM models were implemented with Tensorflow [25] as backend. The area under the ROC-curve (AUC) was chosen as the performance measurement as it is able to measure performance reliably with skewed class distribution [26]. Detailed information about the models and nested cross-validation are provided in Appendix 1.

Next, the best performing model—FastText—was optimized with fivefold cross-validation. The whole dataset was used in optimization to capture as much information as possible for the explanations. In total 225 hyperparameter combinations were tested and hyperparameters with the best mean AUC were chosen to be the parameters of the final explanatory model. The final model was trained with 90% of data and the remaining 10% was used in the explanatory analysis described below with parameters found in hyperparameter optimization. Detailed information about hyperparameter optimization is provided in Appendix 2.

As a third step, the mentioned “explainable XAI” technique LIME was used to extract the importance of words relative to each prediction. The LIME package module used LimeTextExplainer with default parameters except top_labels was set to 1, num_feature to a number of tokens per sample, and num_samples was set to 10,000. 10% of the tokens with positive coefficients were used as keywords. In addition, if the keywords were next to each other, they were combined to be keyphrases to retain the semantic information.

Finally, a manual analysis of the extracted keywords and keyphrases was performed. The evaluation was done to understand whether the algorithm’s results are clinically understandable and relevant. 80 patients were randomly chosen, 20 patients from four different scenarios: subsequent or non-subsequent event for correct or incorrect model predictions. Domain experts evaluated, if they agreed with the result of the algorithm, if the key words found by the algorithm were relevant and if the result of the algorithm was justifiable by the narrative texts of ePCR using a three-class scale: 1 = I disagree, 2 = unclear/more text is needed, 3 = I agree.

The cases were analyzed independently by two researchers (JP and HR). For the cases where the assessments differed (n = 15, 19.5%), a third evaluator, TI, provided a third independent evaluation and these cases were discussed until consensus was reached. Inductive content analyses, which allow categorization and frequency calculation of the words, phrases and expressions were also used as part of the manual evaluation [27].

## Results

**Fig. 4:**
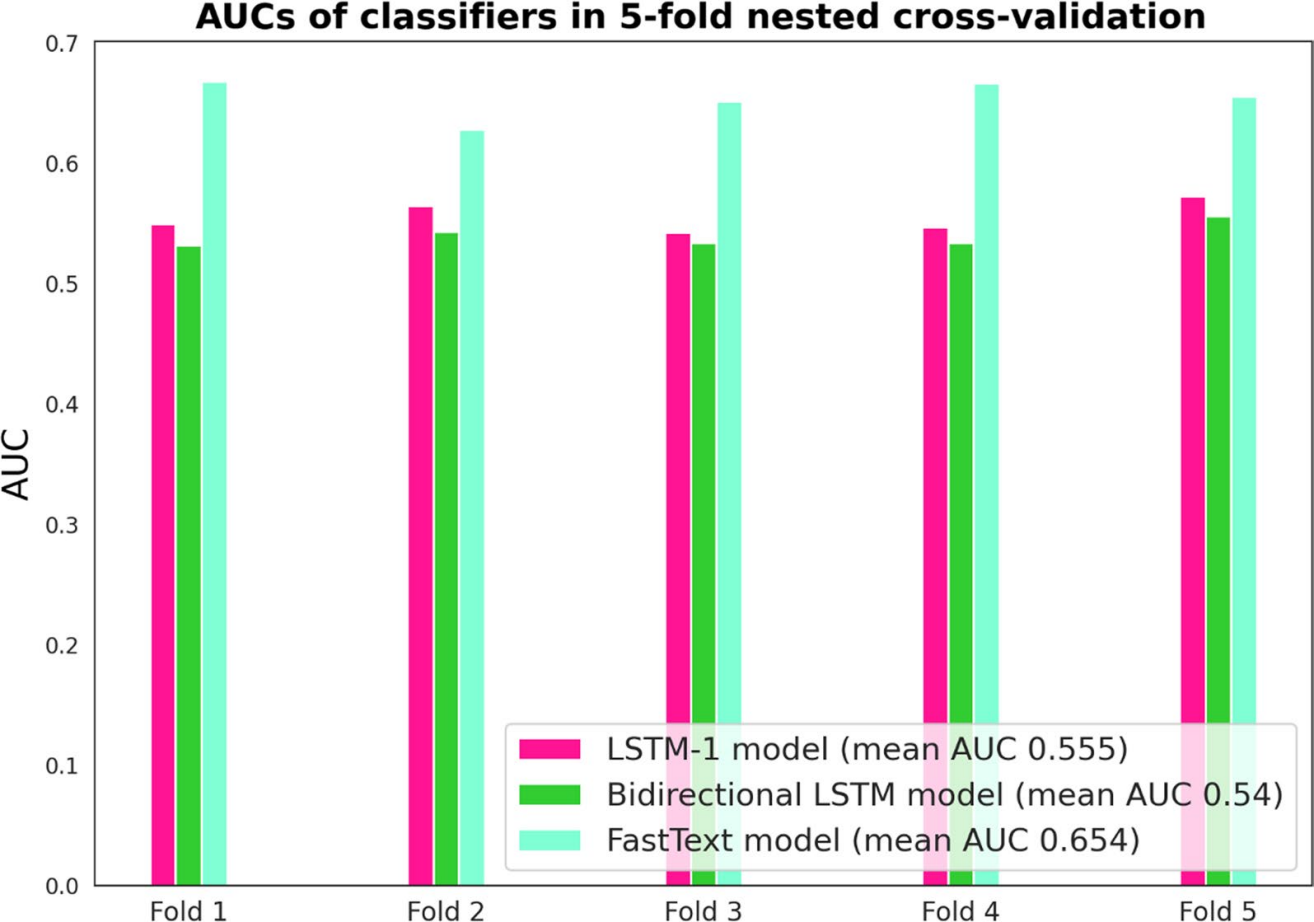
Performance of classifiers in nested cross validation

The text classification model based on FastText performed best according to the nested cross-validation, with a mean AUC of 0.654 (Fig. 4). Best mean AUC (0.662) in hyperparameter optimization was obtained with parameters 'epoch' = 20, 'lr' = 0.1, and 'wordNgrams' = 5.

Manual evaluation showed that the results of the algorithm were clinically relevant. Also, narrative texts of ePCRs gave clues of the algorithm’s results. The extracted keywords by the model were partly irrelevant and manually challenging to identify and understand. For example, there were many conjunctions like “or”, “and”, “no”, “with” and “if”. The most understandable keywords were “tomorrow to health center” or “morning to ED”, and non-specific complaints like “malaise”. Overall, in the cases where the patient had a subsequent event and the model also predicted it, the manual evaluation showed the highest scores (Table 1).

**Table 1:** The study group’s manual evaluation of the algorithm (1 = disagree, 2 = unclear, 3 = agree)

	I agree with algorithm	The key words are relevant	The text gives clues of the algorithm’s result
The patient had subsequent event and the model predicted there will be one (n = 17)	1 = 0% (n = 0) 2 = 23.5% (n = 4) 3 = 76.5% (n = 13)	1 = 41.2% (n = 7) 2 = 17.7% (n = 3) 3 = 41.2% (n = 7)	1 = 0% (n = 0) 2 = 29.4% (n = 5) 3 = 70.6% (n = 12)
The patient had subsequent event, but the model did not predict one (n = 20)	1 = 55.0% (n = 11) 2 = 15.0% (n = 3) 3 = 30.0% (n = 6)	1 = 90.0% (n = 18) 2 = 10.0% (n = 2) 3 = 0% (n = 0)	1 = 40.0% (n = 8) 2 = 30.0% (n = 6) 3 = 30.0% (n = 6)
The patient didn’t have subsequent event, but the model predicted that there will be one (n = 20)	1 = 20.0% (n = 4) 2 = 20.0% (n = 4) 3 = 60.0% (n = 12)	1 = 50.0% (n = 10) 2 = 25.0% (n = 5) 3 = 25.0% (n = 5)	1 = 10.0% (n = 2) 2 = 25.0% (n = 5) 3 = 65.0% (n = 13)
The patient didn’t have subsequent event and the model did not predict one (n = 20)	1 = 15.0% (n = 3) 2 = 40.0% (n = 8) 3 = 45.0% (n = 9)	1 = 95.0% (n = 19) 2 = 5.0% (n = 1) 3 = 0% (n = 0)	1 = 5.0% (n = 1) 2 = 35.0% (n = 7) 3 = 60.0% (n = 12)

Based on the content analyses, three categories were found to predict subsequent events after a non-conveyance decision. 4 in 5 of the subsequent event cases, EMS care providers and the patients had an agreement that the patient will visit primary health care facility or ED next or following days after the initial non-conveyance mission. There were also many who did not go even if they were instructed. The most frequent signs and symptoms as predictors were musculoskeletal-, infection-related and non-specific complaints. There were also some frequent callers with minor problems. Psychological symptoms were common predictors, but the model did not predict them very well (Table 2). In 18.2% (14/77) of the cases, the narrative texts of ePCR were very short. Over a third (5/14) of these briefly documented cases led to subsequent events. Moreover, there were three other cases, where subsequent events seemed inevitable, but these patients did not have it. The model missed four of these eight subsequent events. There were also some random factors like a great number of diseases or homecare providers’ or relatives’ anxiety, which could be related to subsequent events as well.

**Table 2:** Predictors of subsequent events

*The patient had subsequent event and the model predicted there will be one (n = 17)*		
The study group agree with model there will be subsequent event (n = 17)		
Signs/symptoms	n	%
Musculoskeletal symptoms	6	35.3
Infection	3	17.6
Non-specific complaints	2	11.8
Abdominal pain	2	11.8
High blood pressure	1	5.9
Ear pain	1	5.9
Nasal bleeding	1	5.9
Frequent caller with minor problem	1	5.9
The agreement with the patient/guidance to visit primary health care or ED next or following days 76.5% of the cases (13/17)		

*The patient had subsequent event, but the model did not predict one (n = 20)*		
The study group agree with model there won't be subsequent event (n = 7)		
Signs/symptoms	n	%
Frequent caller with minor problem	3	15.0
Fall due to alcohol misuse	1	5.0
Urinary catheter blocked	1	5.0
Psychological symptom	1	5.0
Allergic reaction	1	5.0
The agreement with the patient/guidance to visit primary health care or ED next or following days 0% of the cases (0/7)		

The study group disagree with model there won't be subsequent event (n = 13)		
Signs/symptoms	n	%
Psychological symptom	5	25.0
Musculoskeletal symptoms	3	15.0
Non-specific complaints	2	10.0
Convulsion	1	5.0
Violence/assault	1	5.0
Nausea	1	5.0
The agreement with the patient/guidance to visit primary health care or ED next or following days 76.9% of the cases (10/13)		

*The patients didn’t have subsequent event, but the model predicted that there will be one (n = 20)*		
The study group agree with model there will be subsequent event (n = 18)		
Signs/symptoms	n	%
Musculoskeletal symptoms	11	55.0
Infection	2	10.0
Breathing trouble	2	10.0
Psychological symptom	1	5.0
High blood pressure	1	5.0
Diarrhoea	1	5.0
The agreement with the patient/guidance to primary health care or ED next or following days 66.7% of the cases (12/18)		

The study group disagree with model that there will be subsequent event (n = 2)		
Signs/symptoms	n	%
Musculoskeletal symptoms	1	5.0
Breathing trouble	1	5.0
The agreement with the patient/guidance to visit primary health care or ED next or following days 0% of the cases (0/2)		

*The patients didn’t have subsequent event and the model did not predict one (n = 20)*		
The study group agree with model there won’t be subsequent event (n = 15)		
Signs/symptoms	n	%
Psychological symptom	4	20.0
Chest pain	3	15.0
Abdominal pain	2	10.0
Fall (elderly with minor conditions)	2	10.0
Musculoskeletal symptoms	1	5.0
Fall due to alcohol misuse	1	5.0
Toxic effect of carbon monoxide	1	5.0
Bicycle accident	1	5.0
The agreement with the patient/guidance to visit primary health care or ED next or following days 0% of the cases (0/15)		

The study group disagree with model there won’t be subsequent event (n = 5)		
Signs/symptoms	n	%
Non-specific complaints	2	10.0
Psychological symptom	1	5.0
Musculoskeletal symptoms	1	5.0
Symptoms of apoplexia	1	5.0
The agreement with the patient/guidance to visit primary health care or ED next or following days 60.0% of the cases (3/5)		

## Discussion

The main findings are as follows. Machine learning (FastText-model, AUC 0.654) seems promising at predicting subsequent events after EMS non-conveyance decisions. In case of subsequent events, many of these patients were guided to visit primary health care facilities or ED next or following days after non-conveyance. Musculoskeletal-, infection-related- and non-specific complaints were the most frequent signs and symptoms as subsequent event predictors.

To the best of our knowledge, this is the first study where machine learning is used to search for predictors from narrative texts of ePCR in the context of EMS’ non-conveyance. Our study demonstrated that the FastText-model performed better than the two other LSTM-based neural network text classification models. This demonstrates that non-linear classifiers do not generalize well to this data. This is likely due to the complexity of the task and the limited training data. The manual evaluation indicated that the predictions made by the FastText-model were understandable and clinically important, which increases the reliability of this study even if the keywords were partly unclear.

While these results are promising, the prediction of the subsequent event from an individual narrative text of ePCR in this context is a hard task even for the comprehensively optimized FastText model. This indicates the complexity of the original task and limited opportunities for observations that the EMS care providers face.

As mentioned earlier, 90% of the data was used for the training of the model and 10% was used for the explanatory analysis. It is likely that prediction performance may increase with more data. Thus, more data and additional studies are needed. In addition, leveraging transformer-based models pre-trained on Finnish electronic health records may have increased the performance [28], but as their large size in practice prevents the use of nested cross-validation in performance estimation, transformers based models were excluded from this study.

The keywords extracted by the model and the manual analyses showed that a number of the subsequent events were planned by EMS personnel. Therefore it seems that after assessment and treatment EMS personnel evaluate that these patients do not need a doctor immediately but a subsequent visit for example in the next morning is appropriate. Related to this, previous studies have shown that there is a correlation between increased likelihood of non-conveyance and the following factors: non-urgent missions, EMS arrival time in the evening or at night, and the destination being in a rural area [3, 5], and the following subsequent visits in primary health care [8]. Furthermore, EMS patients are often in good condition [3, 8]. This indicates that the resources were correctly directed by EMS and unnecessary conveyance to the ED was avoided. In many areas, access to primary health care is limited and EMS is the only 24/7 health care service [4]. A Finnish study indicated that after the evaluation almost half the non-conveyed patients were instructed to contact primary health care during daytime [5]. The role of EMS has changed to include more non-critical patients instead of traditional high risk patient groups [3] like “first hour quintet” [29]. On the other hand, unnecessary EMS missions are discussed globally [30] and our study demonstrated there were many patients who skipped the subsequent visit to the doctor despite the guidance by EMS. However, the guidance by EMS indicates that EMS care providers were concerned about the patient's condition. It seems that the subsequent visit can wait for a while, but further contact is anyway required.

Acute musculoskeletal symptoms were the most common signs and symptoms, which predicted the subsequent events after the patients were discharged at the scene. Understandably, these problems are often non-urgent and therefore non-conveyance decisions and the instructions to visit the doctor are justified. Moreover, there were some frequent callers with minor problems. Other studies have found that one in three EMS patients is a frequent caller [5, 31]. On the other hand, infections and non-specific complaints were common predictors as well. Previous studies have reported that infections like sepsis are challenging to identify [32] and non-specific complaints predict many subsequent events in both the contexts of prehospital emergency care and EDs [33–35]. This raises the question of whether the non-conveyance decision of these patients is justified, even if many of these subsequent events were planned before as well. However, there should be a balance between safety margins and wasting limited resources. In addition, psychological symptoms were also common, but these patients were challenging for the algorithm to find, maybe due to varied signs and symptoms.

Based on our results, the narrative texts of ePCRs were very short in one of five missions and many of these led to a subsequent event. The model missed some of these cases probably due to limited information. More studies are needed to address the reasons for the inadequate documentation. Previous studies have reported new guidelines [36], checklists [37], educational interventions [38] and body-worn cameras to improve EMS documentation instead of short-term memories, for example [39]. Finally, incomplete documentation is a major risk for subsequent events in prehospital emergency care [40, 41].

### Limitations

This study has limitations. The excluded patients [3], the challenges of exact time of the ED visits, the fact that the subsequent visits to primary health care includes chronic disease monitoring and generalizability of the rates of subsequent event and safety factors, were described previously [8].

In this study, the data were labeled in two groups, non-subsequent event and subsequent event. Thus, all subsequent events were thought to be equivalent. The data set for the machine learning analyses was small (90% for the model training and 10% for the explanatory analysis), and the number of subsequent events was relatively small. Therefore, when the eighty missions were randomly chosen for the clinical analyses, there were only seventeen cases to represent the combination of subsequent events and correct predictions by the model.

The performance of FastText was better than random, but still, the performance could be better. As we used all available data for optimization, the final explanatory analysis is slightly optimistic (0.008 higher AUC in the explanatory analysis compared to nested cross-validation results). LIME explains predictions in the local neighborhood of samples, thus it is hard to draw global conclusions from explanations even when a large number of sample predictions have been explained. In addition, the LIME explanation may be an inaccurate representation of the original prediction and explaining predictions of an uncertain model may give a biased estimation of true phenomenon [42].

It is notable that the narrative texts of ePCRs are difficult to analyze computationally, but also manually. The texts are short, there are several abbreviations used, either formal or informal ones [40]. Further, the Finnish language is challenging from a natural language processing perspective due to its many cases and inflections. In some cases, the patients had multiple types of signs and symptoms, but in the narrative texts, only the main one, or some combination, were described. In this study, only the narrative texts were analyzed, but in future, the texts can be combined to structured data like physiological parameters. Moreover, it is likely that there are factors related to the EMS non-conveyance decisions and the following subsequent events that were not found in this study. For instance, EMS care providers’ tacit knowledge is a typical thing that goes unrecorded.

## Conclusions

This study shows that machine learning in the form of text classification can be used to predict subsequent events from narrative texts of ePCR after EMS non-conveyance decisions. It is notable that these subsequent events do not necessary mean that patient safety is jeopardized. This study shows that many subsequent visits to primary health care or EDs were planned beforehand by EMS personnel. This indicates reasonable use of limited resources to decrease ED crowding. However, more research is needed. The machine learning model could be tested for each subsequent event type separately and exclude planned subsequent events in order to find out the harmful subsequent events, where EMS non-conveyance puts patient safety at risk.

## Appendices

### Appendix 1: Details of models and hyperparameters tested in nested cross validation. Underlined hyperparameters were tuned

A Sequential LSTM model was implemented with Tensorflow (version 2.2.0). Model performance was monitored with a validation set (10% of training set of each cross-validation fold) to avoid overfitting. Additionally, EarlyStopping with patience 20 and ReduceLROnPlateau with factor 0.5 and patience 2 callbacks were used. Maximum number of epochs was 10 and Adam (learning_rate = 0.001, beta_1 = 0.9, beta_2 = 0.999, epsilon = 1e−07) was used as an optimizer and BinaryCrossentropy was used as a loss function. Data was first transformed to numerical format with Tensorflow datasets (version 1.2.0) module SubwordTextEncoder (vocabulary size 9949), then padded to maximum length of a batch. Batch size was set to 50. 5 different hyperparameter combinations were used in each inner fold. Following parameters were tested where first number is the parameter combination number, second is number of units and third is the drop rate: {"1": [100, 0.1], "2": [300, 0.2], "3": [450, 0.3], "4": [200, 0.2], "5": [450, 0.4]}.

**Table:** 

Layers and untuned parameters	Hyperparameters	Values
Embedding(input_dim = 9949 + 1,output_dim = 200,mask_zero = True)	–	–
Dropout	drop_rate	0.1, 0.2, 0.3, 0.4
LSTM(dropout = 0.2,recurrent_dropout = 0.2,activation = 'sigmoid')	Units	100, 200, 300, 450
Dense(units = 1, activation = 'sigmoid')	–	–

A Sequential Bidirectional-LSTM model was implemented with Tensorflow (version 2.2.0). Model performance was monitored with a validation set (10% of training set of each cross-validation fold) to avoid overfitting. Additionally, EarlyStopping with patience 20 and ReduceLROnPlateau with factor 0.5 and patience 2 callbacks were used. Maximum number of epochs was 10 and Adam (learning_rate = 0.001, beta_1 = 0.9, beta_2 = 0.999, epsilon = 1e−07) was used as an optimizer and BinaryCrossentropy was used as a loss function. Data was first transformed to numerical format with Tensorflow datasets (version 1.2.0) module SubwordTextEncoder (vocabulary size 9949), then padded to maximum length of a batch. Batch size was set to 50. 5 different hyperparameter combinations were used in each inner fold. Following parameters were tested where first number is the parameter combination number, second is drop rate of first dropout layer, third is the number of units in bidirectional-LSTM, fourth is drop rate of second dropout layer, and fifth is number of units of LSTM layer: {“1”: [0.1, 200, 64, 0.1], “2”: [0.2, 200, 100, 0.2], “3”: [0.3, 300, 64, 0.2], “4”: [0.2, 200, 64, 0.2], “5”: [0.2, 300, 200, 0.3]}.

**Table:** 

Layers and untuned parameters	Hyperparameters	Values
Embedding(input_dim = 9949 + 1,output_dim = 200,mask_zero = True)	–	–
Dropout	Drop_rate	0.2, 0.3
Biderctional-LSTM(dropout = 0.2,recurrent_dropout = 0.2,activation = 'sigmoid',return-sequences = True)	Units	200, 300
LSTM(dropout = 0.2,recurrent_dropout = 0.2,activation = 'sigmoid')	Units	64, 100, 200
Dropout	Drop_rate	0.1, 0.2, 0.3
Dense(units = 1, activation = 'sigmoid')	–	–

The FastText model was trained with the train_supervised() method with default parameters except ones in table below. Following parameters were tested where first number is the parameter combination number, second is learning rate, third is number of epochs and fourth is max length of word ngram: {"1": [0.6,40,5], "2": [0.3,40,3], "3": [0.7,30,4], "4": [0.2,50,5], "5": [0.4,40,2]}

**Table:** 

Hyperparameter	Value
lr	0.2, 0.3, 0.4, 0.6, 0.7
epoch	40, 50
wordNgrams	2, 3, 5

### Appendix 2: Hyperparameters of FastText tested in optimization. Underlined hyperparameters were tuned

Parameters of FastText. All parameter combinations in the presented range were tested. Other than hyperparameters described below were default.

**Table:** 

Hyperparameter	Value
lr	0.1, 0.2, 0.3, 0.4, 0.5, 0.6, 0.7, 0.8, 0.9
epoch	20, 30, 40, 50, 60
wordNgrams	1, 2, 3, 4, 5

## Data Availability

The data of this study is not available due to patients’ privacy and research permissions.

## Abbreviations

EMS: Emergency medical services
ED: Emergency Department
LIME: The local interpretable model-agnostic explanations
